# Supplementary figures and images for: Evaluating extraction methods to study canine urine microbiota
Source: PLoS One. 2021 Jul 9;16(7):e0253989. doi: 10.1371/journal.pone.0253989 (PMC8270191; doi:10.1371/journal.pone.0253989)

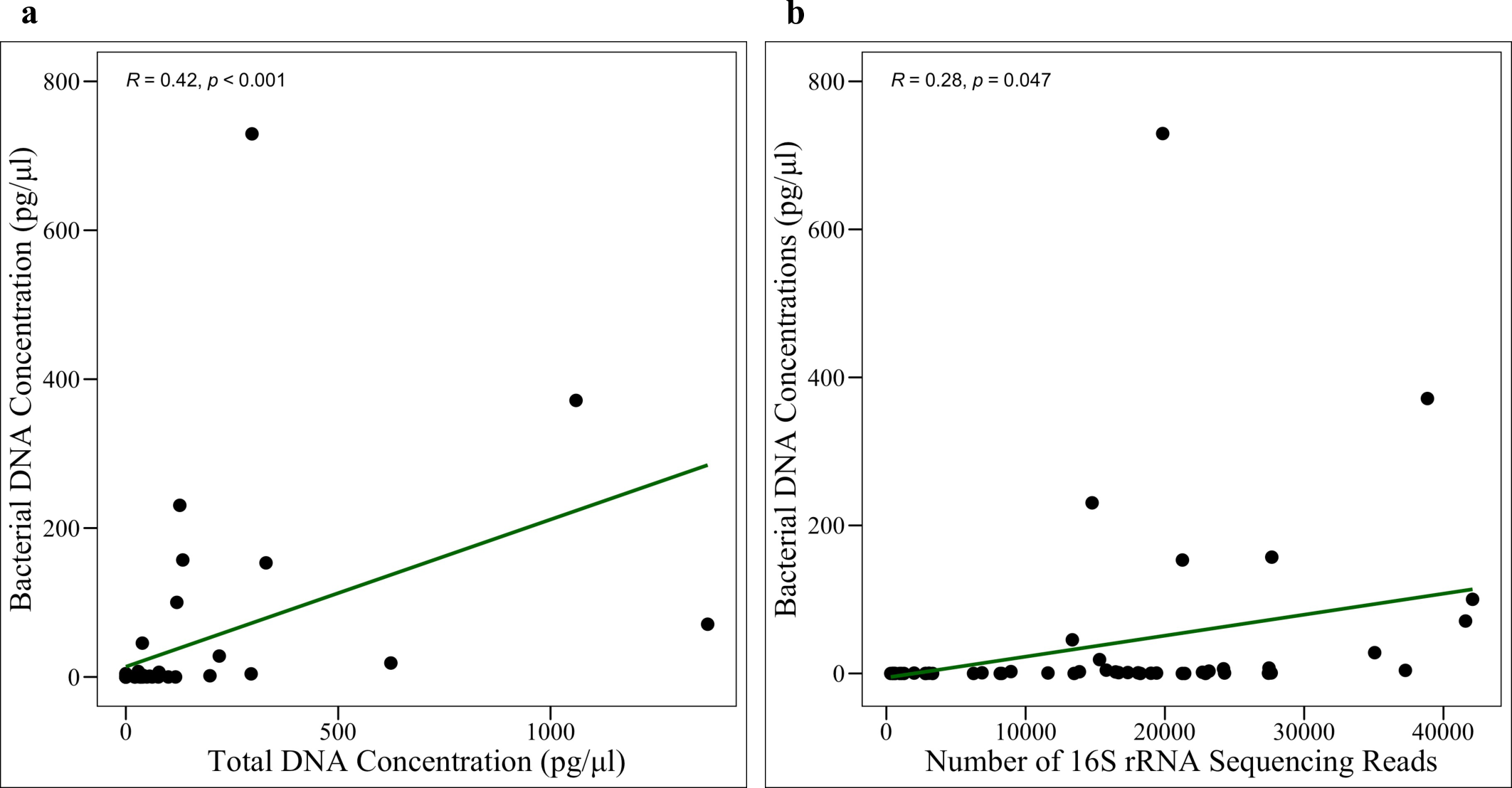

Supplement: S1 Fig — Correlation between (a) total and bacterial DNA concentrations and (b) the number of 16S rRNA sequencing reads and bacterial DNA concentrations. (TIF) [file pone.0253989.s001.tif]

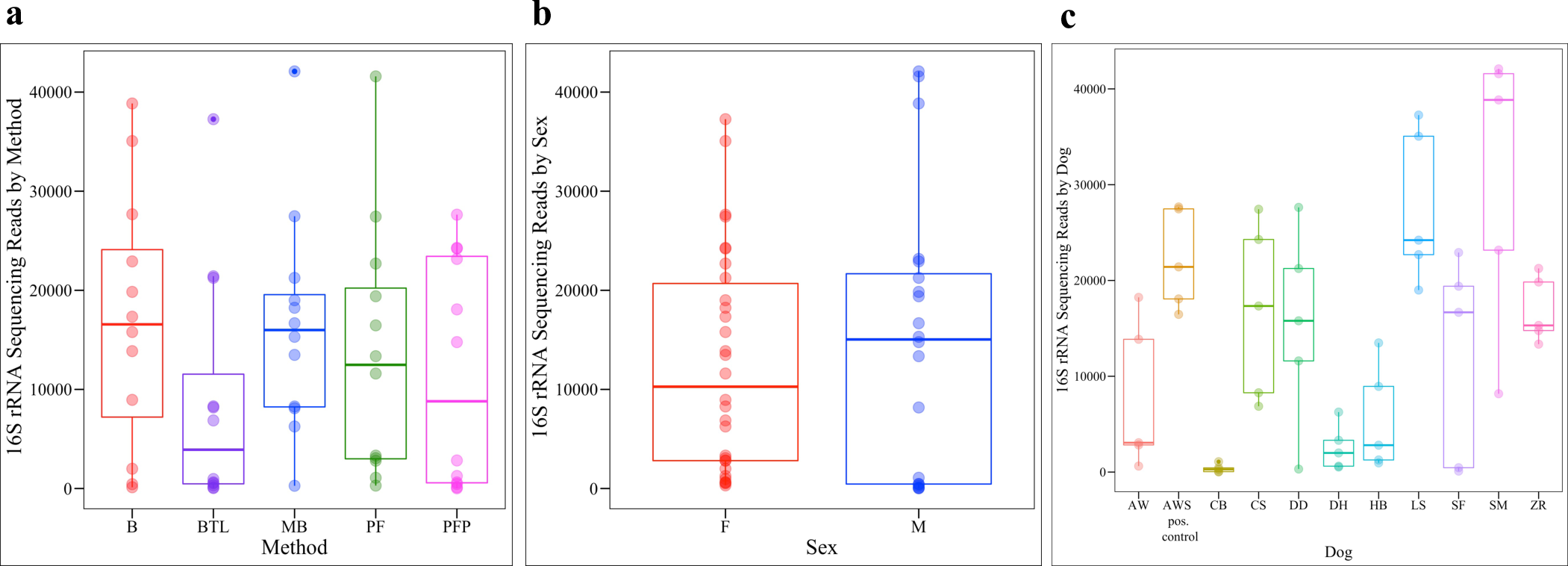

Supplement: S2 Fig — The number of 16S reads per sample was compared by (a) extraction method, (b) sex, and (c) dog. There were no significant differences in the number of reads by method (Kruskal-Wallis, p = 0.378) and sex (Kruskal-Wallis, p = 0.937) but there was a significant difference in the number of reads by dog (Kruskal-Wallis, p = <0.00001). (TIF) [file pone.0253989.s002.tif]

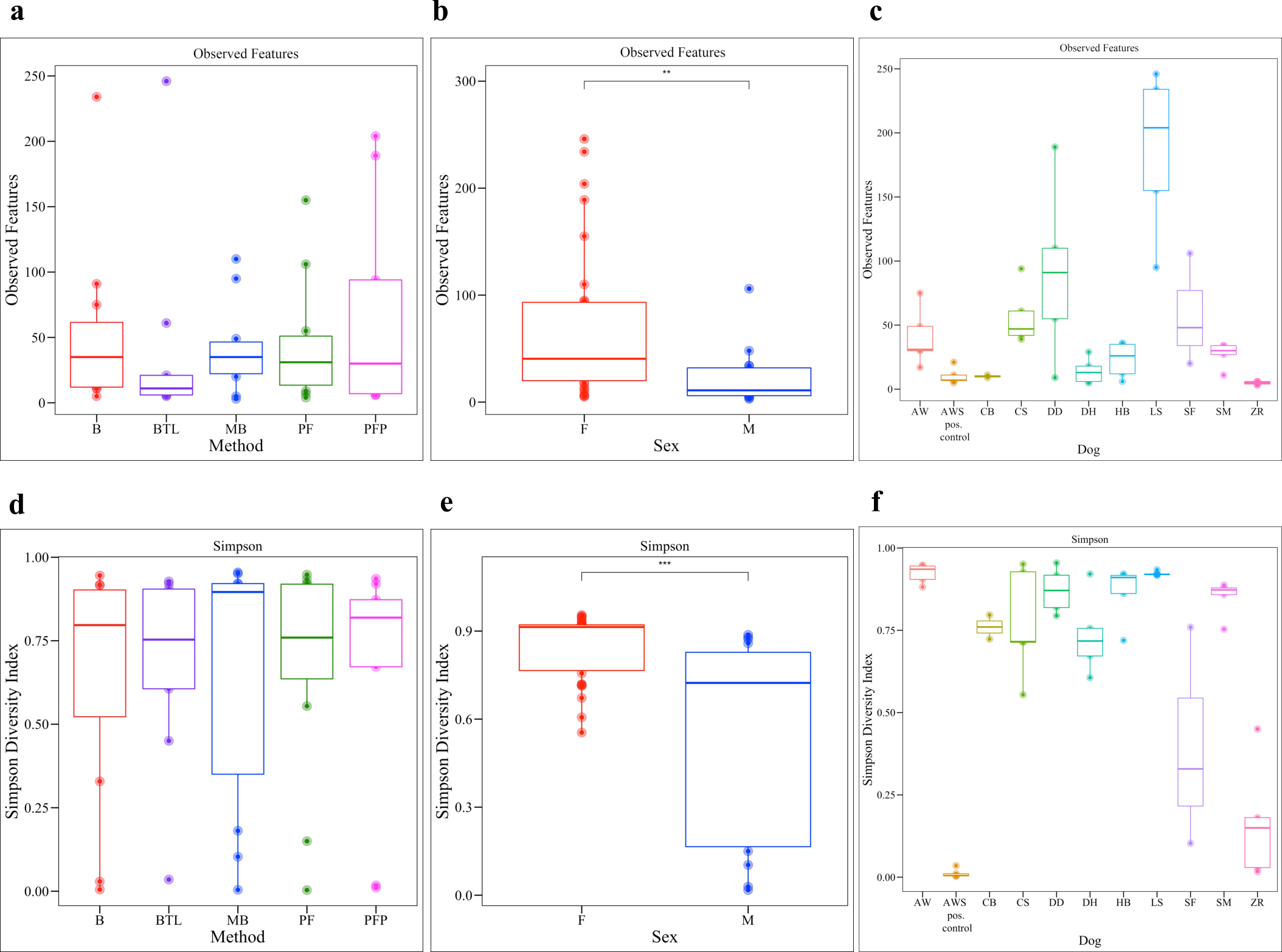

Supplement: S3 Fig — Observed Features and the Simpson index were used to compare microbial diversity by extraction method (a, d), sex (b, e), and dog (c, f). Microbial diversity did not differ by extraction method (Kruskal-Wallis; Observed Features, p = 0.751; Simpson Index, p = 0.872) but did differ by dog (Kruskal-Wallis; Observed Features, p = < 0.001; Simpson Index, p = < 0.001). For all statistically significant pairwise comparisons by dog, see S6 Table. Females exhibited significantly higher microbial diversity than males (Kruskal-Wallis: Observed Features, p = 0.005; Simpson, p = < 0.001). B = Bacteremia, BTL = Blood Tissue with Lysozyme, MB = Magnetic Beads, PF = PowerFecal®, PFP = PowerFecal®Pro, F = Female, M = Male. (TIF) [file pone.0253989.s003.tif]

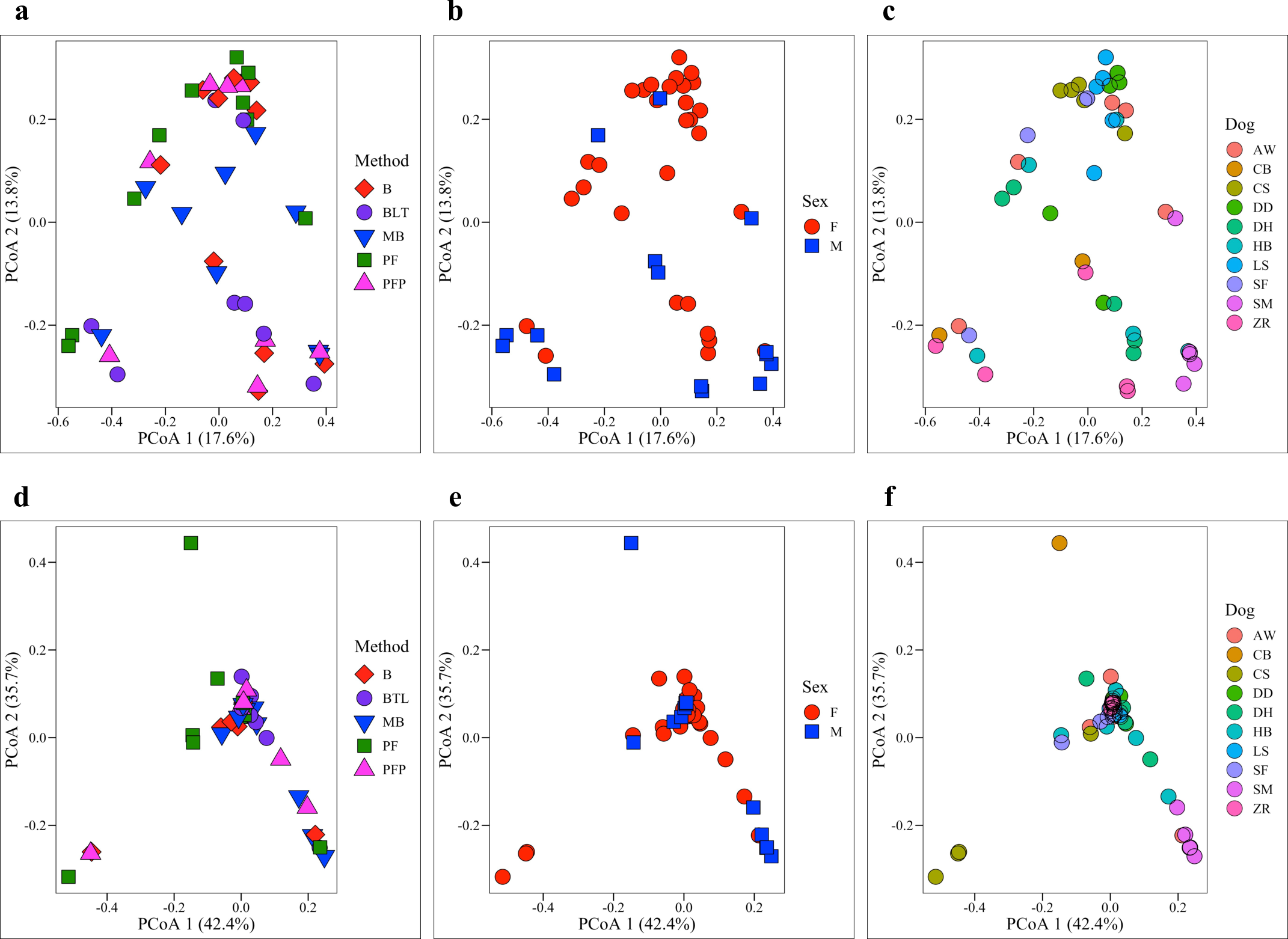

Supplement: S4 Fig — Unweighted (a-c) and Weighted UniFrac matrices (beta-diversity) (d-f) comparing microbial composition by (a, d) extraction method, (b, e) sex, (c, f) and dog. Microbial composition did not differ significantly by extraction method (Kruskal-Wallis: Unweighted UniFrac, p = 0.539; Weighted UniFrac, p = 0.743) but did differ significantly by sex (Kruskal-Wallis: Unweighted UniFrac, p = 0.003; Weighted UniFrac, p = 0.03) and dog (Kruskal-Wallis: Unweighted UniFrac, p = < 0.001; Weighted UniFrac, p = < 0.001) (TIF) [file pone.0253989.s004.tif]

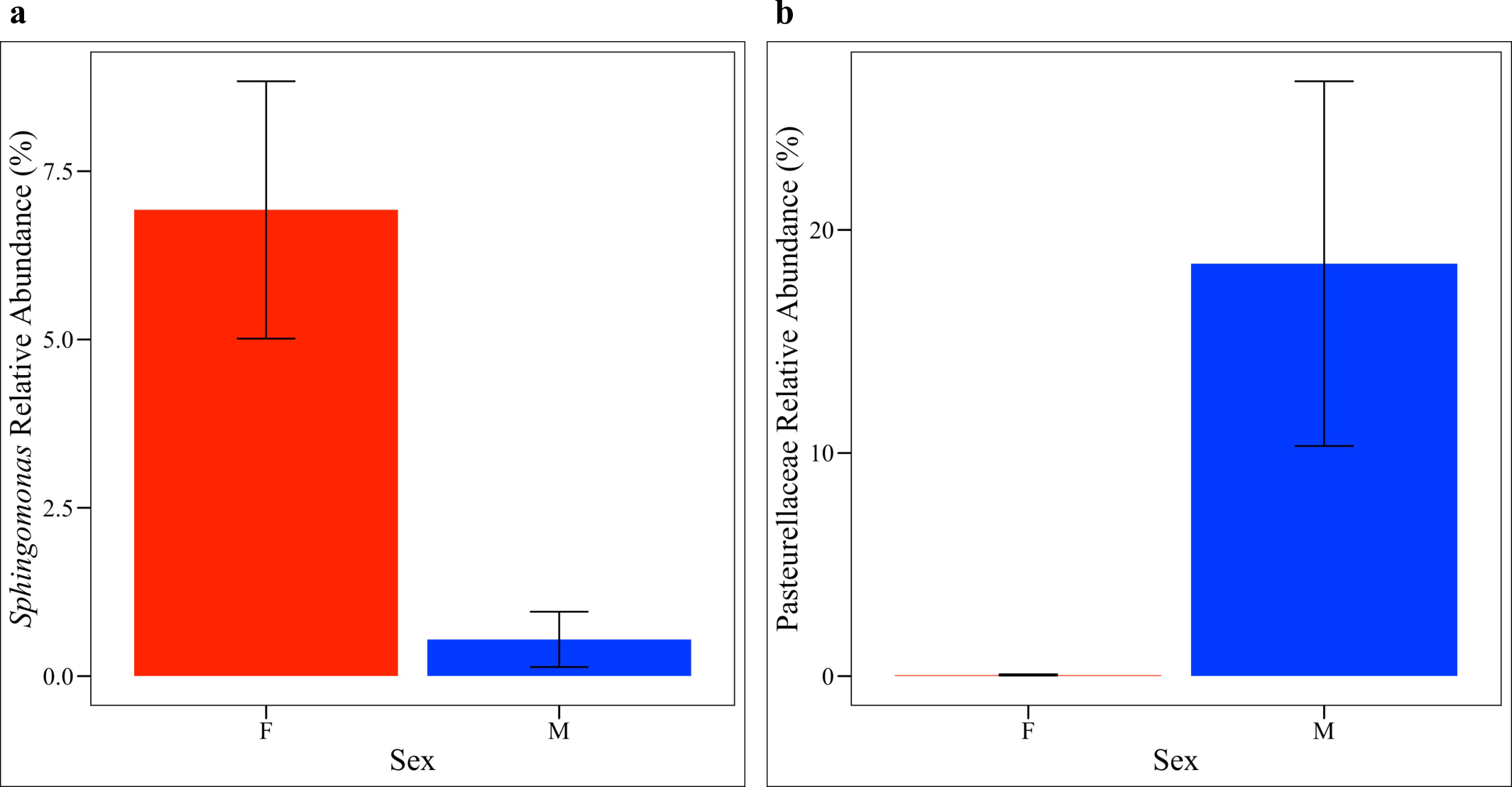

Supplement: S5 Fig — (a) Females had significantly greater relative abundances of Sphingomonas (ANCOM, W = 579) while (b) males had significantly greater relative abundances of Pasteurellaceae bacterium canine oral taxon 272 (ANCOM, W = 596). (TIF) [file pone.0253989.s005.tif]
