# Supplementary material for: Evaluating extraction methods to study canine urine microbiota
Source: PLoS One. 2021 Jul 9;16(7):e0253989. doi: 10.1371/journal.pone.0253989 (PMC8270191; doi:10.1371/journal.pone.0253989)
Supplement: S1 Table — Sex, age, and breed of dogs enrolled in this study. (DOCX) [file pone.0253989.s006.docx]

**Table S1*.* Dog Metadata**. Sex, age, and breed of dogs enrolled in this study.

| **Dog ID** | **Sex** | **Spayed / Neutered?** | **Age (years)** | **Breed** |
| --- | --- | --- | --- | --- |
| AW | F | Y | 10 | Pit Bull / Boxer Mix |
| CB | M | Y | 2 | Golden Retriever / Border Collie |
| CS | F | Y | 1.5 | German Shepard / Belgian Malinois |
| DD | F | Y | 5 | Husky / Cattle Dog |
| DH | F | Y | 4 | Mastiff / St. Bernard Mix |
| HB | F | Y | 1.3 | Border Collie Mix |
| LS | F | N | 0.75 | Golden Retriever |
| SF | M | Y | 6 | Great Pyrenees |
| SM | M | N | 0.6 | Golden Retriever |
| ZR | M | Y | 1 | Pit Bull Mix |
