# Supplementary material for: Evaluating extraction methods to study canine urine microbiota
Source: PLoS One. 2021 Jul 9;16(7):e0253989. doi: 10.1371/journal.pone.0253989 (PMC8270191; doi:10.1371/journal.pone.0253989)
Supplement: S3 Table — P-values based on Wilcoxon Rank Sum Tests for total DNA concentrations using 1000 permutations and False Discovery Rate corrections. There were no statistically significant pairwise comparisons. (DOCX) [file pone.0253989.s008.docx]

**Table S3 *–* Total DNA Concentration Pairwise Comparisons by Dog.** P-values based on Wilcoxon Rank Sum Tests for total DNA concentrations using 1000 permutations and False Discovery Rate corrections. There were no statistically significant pairwise comparisons.

|  | AW | AWS | CB | CS | CTL | DD | DH | HB | LS | SF | SM |
| --- | --- | --- | --- | --- | --- | --- | --- | --- | --- | --- | --- |
| AWS | 0.25 | - | - | - | - | - | - | - | - | - | - |
| CB | 0.29 | 0.48 | - | - | - | - | - | - | - | - | - |
| CS | 1.00 | 0.20 | 0.25 | - | - | - | - | - | - | - | - |
| CTL | 0.52 | 0.11 | 0.11 | 0.52 | - | - | - | - | - | - | - |
| DD | 0.52 | 0.11 | 0.11 | 0.52 | - | - | - | - | - | - | - |
| DH | 0.88 | 0.25 | 0.37 | 0.70 | 0.29 | 0.29 | - | - | - | - | - |
| HB | 1.00 | 0.25 | 0.29 | 1.00 | 0.52 | 0.52 | 0.88 | - | - | - | - |
| LS | 0.15 | 0.38 | 0.25 | 0.15 | 0.11 | 0.11 | 0.20 | 0.15 | - | - | - |
| SF | 0.29 | 0.98 | 0.48 | 0.15 | 0.11 | 0.11 | 0.29 | 0.25 | 0.29 | - | - |
| SM | 0.29 | 0.77 | 0.62 | 0.29 | 0.20 | 0.20 | 0.39 | 0.29 | 1.00 | 0.62 | - |
| ZR | 0.11 | 0.25 | 0.11 | 0.11 | 0.11 | 0.11 | 0.11 | 0.11 | 0.45 | 0.13 | 0.90 |
