# Supplementary material for: Evaluating extraction methods to study canine urine microbiota
Source: PLoS One. 2021 Jul 9;16(7):e0253989. doi: 10.1371/journal.pone.0253989 (PMC8270191; doi:10.1371/journal.pone.0253989)
Supplement: S4 Table — P-values based on Wilcoxon Rank Sum Tests for total DNA concentrations using 1000 permutations and False Discovery Rate corrections. There were no statistically significant pairwise comparisons. (DOCX) [file pone.0253989.s009.docx]

**Table S4 *–* Bacterial DNA concentration pairwise comparisons by dog.** P-values based on Wilcoxon Rank Sum Tests for total DNA concentrations using 1000 permutations and False Discovery Rate corrections. There were no statistically significant pairwise comparisons.

|  | AW | AWS | CB | CS | CTL | DD | DH | HB | LS | SF | SM |
| --- | --- | --- | --- | --- | --- | --- | --- | --- | --- | --- | --- |
| AWS | 0.341 | - | - | - | - | - | - | - | - | - | - |
| CB | 0.171 | 0.103 | - | - | - | - | - | - | - | - | - |
| CS | 0.610 | 0.405 | 0.103 | - | - | - | - | - | - | - | - |
| CTL | 0.171 | 0.103 | - | 0.103 | - | - | - | - | - | - | - |
| DD | 0.722 | 0.341 | 0.171 | 1.000 | 0.171 | - | - | - | - | - | - |
| DH | 0.497 | 0.139 | 0.530 | 0.171 | 0.530 | 0.293 | - | - | - | - | - |
| HB | 0.567 | 0.241 | 0.530 | 0.410 | 0.530 | 0.497 | 1.000 | - | - | - | - |
| LS | 0.119 | 0.778 | 0.086 | 0.094 | 0.086 | 0.171 | 0.094 | 0.107 | - | - | - |
| SF | 1.000 | 0.221 | 0.251 | 0.410 | 0.251 | 0.502 | 0.562 | 0.722 | 0.107 | - | - |
| SM | 0.497 | 0.722 | 0.171 | 0.556 | 0.171 | 0.497 | 0.302 | 0.302 | 0.717 | 0.497 | - |
| ZR | 0.086 | 0.164 | 0.086 | 0.086 | 0.086 | 0.086 | 0.086 | 0.086 | 0.109 | 0.103 | 0.824 |
