# Supplementary material for: Evaluating extraction methods to study canine urine microbiota
Source: PLoS One. 2021 Jul 9;16(7):e0253989. doi: 10.1371/journal.pone.0253989 (PMC8270191; doi:10.1371/journal.pone.0253989)
Supplement: S5 Table — We employed both the frequency and prevalence methods in the R package decontam v.1.10.0 to identify putative contaminant ASVs. In total, 28 ASVs were identified and excluded from our analysis as contaminants. (DOCX) [file pone.0253989.s010.docx]

**Table S5 – ASVs identified as contaminants.** We employed both the frequency and prevalence methods in the R package decontam v.1.10.0 to identify putative contaminant ASVs. In total, 28 ASVs were identified and excluded from our analysis as contaminants.

| ASV | Method |
| --- | --- |
| D_0__Bacteria;D_1__Proteobacteria;D_2__Alphaproteobacteria;D_3__Caulobacterales;D_4__Caulobacteraceae;D_5__Brevundimonas | Frequency |
| D_0__Bacteria;D_1__Bacteroidetes;D_2__Bacteroidia;D_3__Sphingobacteriales;D_4__Sphingobacteriaceae;D_5__ Pedobacter | Frequency |
| D_0__Bacteria;D_1__Bacteroidetes;D_2__Bacteroidia;D_3__Chitinophagales;D_4__Chitinophagaceaee;D_5__ Taibaiella;D_6__Taibaiella coffeisoli | Frequency |
| D_0__Bacteria;D_1__Firmicutes;D_2__Bacilli;D_3__Bacillales;D_4__Bacilliaceae;D_5__Bacillus | Frequency |
| D_0__Bacteria;D_1__Bacteroidetes;D_2__Bacteroidia;D_3__Flavobacteriales;D_4__Weeksellaceae;D_5__ Bergeyella;D_6__Cloacibacterium sp. canine oral taxon 320 | Frequency |
| D_0__Bacteria;D_1__Firmicutes;D_2__Bacilli;D_3__Bacillales;D_4__Staphylococcaceae;D_5__Staphylococcus | Frequency |
| D_0__Bacteria;D_1__Proteobacteria;D_2__Gammaproteobacteria;D_3__Betaproteobacteria;D_4__ Procabacteriaceae;D_5__Procabacter | Frequency |
| D_0__Bacteria;D_1__Firmicutes;D_2__Clostridia;D_3__Clostridiales;D_4__Ruminococcaceae;D_5__ Faecalibacterium | Frequency |
| D_0__Bacteria;D_1__Proteobacteria;D_2__Gammaproteobacteria;D_3__Pseudomonadales;D_4__Moraxellaceae;D_5__Moraxella;D_6__unidentified | Frequency |
| D_0__Bacteria;D_1__Proteobacteria;D_2__Alphaproteobacteria;D_3__Rhizobiales;D_4__Xantobacteraceae;D_5__ Bradyrhizobium | Prevalence |
| D_0__Bacteria;D_1__Bacteroidetes;D_2__Bacteroidia;D_3__Flavobacteriales | Prevalence |
| D_0__Bacteria;D_1__Proteobacteria;D_2__Gammaproteobacteria;D_3__Betaproteobacteriales;D_4__ Methylophilaceae;D_5__Methylotenera | Prevalence |
| D_0__Bacteria;D_1__Bacteroidetes;D_2__Bacteroidia;D_3__Bacteroidales;D_4__Prevotellaceae;D_5__Prevotella 9 | Prevalence |
| D_0__Bacteria;D_1__Proteobacteria;D_2__Gammaproteobacteria;D_3__Betaproteobacteriales;D_4__Burkholderiaceae;D_5__Aquabacterium;D_6__Aquabacterium citratiphium | Prevalence |
| D_0__Bacteria;D_1__Proteobacteria;D_2__Gammaproteobacteria;D_3__Betaproteobacteria;D_4__Burkholderiaceae | Prevalence |
| D_0__Bacteria;D_1__Proteobacteria;D_2__Alphaproteobacteria;D_3__Caulobacterales;D_4__Caulobacteraceae;D_5__Brevundimonas | Prevalence |
| D_0__Bacteria;D_1__Patescibacteria;D_2__ABY1;D_3__Candidatus Megasanikbacteria;D_4__uncultured bacterium;D_5__uncultured bacterium;D_6__uncultured bacterium | Prevalence |
| D_0__Bacteria;D_1__Firmicutes;D_2__Bacilli;D_3__Lactobacillales;D_4__Leuconostocaceae;D_5__Weissella | Prevalence |
| D_0__Bacteria;D_1__Proteobacteria;D_2__Gammaproteobacteria;D_3__Aeromonadales;D_4__Aeromonadaceae;D_5__Aeromonas | Prevalence |
| D_0__Bacteria;D_1__Actinobacteria;D_2__Actinobacteria;D_3__Micrococcales;D_4__Microbacteriaceae | Prevalence |
| D_0__Bacteria;D_1__Proteobacteria;D_2__Gammaproteobacteria;D_3__Betaproteobacteriales;D_4__ Burkholderiaceae;D_5__Acidovorax | Prevalence |
| D_0__Bacteria;D_1__Spirochaetes;D_2__Spirochaetia;D_3__Spirochaetales;D_4__Spirochaetaceae;D_5__ Treponema 2;D_6__Treponema succinifaciens DSM 2489 | Prevalence |
| D_0__Bacteria;D_1__Firmicutes;D_2__Bacilli;D_3__Bacillales;D_4__Staphylococcaceae;D_5__Staphylococcus | Prevalence |
| D_0__Bacteria;D_1__Actinobacteria;D_2__Actinobacteria;D_3__Frankiales;D_4__Geodermatophilaceae;D_5__ Geodermatophilus | Prevalence |
| D_0__Bacteria;D_1__Actinobacteria;D_2__Actinobacteria;D_3__Micrococcaceae;D_5__Micrococcus | Prevalence |
| D_0__Bacteria;D_1__Bacteroidetes;D_2__Bacteroidia;D_3__Bacteroidales;D_4__Bacteroidaceae;D_5__Bacteroides | Prevalence |
| D_0__Bacteria;D_1__Actinobacteria;D_2__Actinobacteria;D_3__Frankiales;D_4__Cryptosporangiaceae;D_5__ Fodinicola;D_6__metagenome | Prevalence |
| D_0__Bacteria;D_1__Proteobacteria;D_2__Alphaproteobacteria;D_3__Caulobacterales;D_4__Caulobacteraceae;D_5__uncultured | Prevalence |
