# Supplementary material for: Evaluating extraction methods to study canine urine microbiota
Source: PLoS One. 2021 Jul 9;16(7):e0253989. doi: 10.1371/journal.pone.0253989 (PMC8270191; doi:10.1371/journal.pone.0253989)
Supplement: S6 Table — P-values resulting from pairwise Wilcoxon Rank Sum Tests for alpha diversity metrics using 1000 permutations and False Discovery Rate corrections. *p < 0.05. (DOCX) [file pone.0253989.s011.docx]

**Table S6** – **Alpha-diversity pairwise comparisons by dog**. P-values resulting from pairwise Wilcoxon Rank Sum Tests for alpha diversity metrics using 1000 permutations

and False Discovery Rate corrections. *p < 0.05

| **Observed OTUs p-values** | | | | | | | | | | | |
| --- | --- | --- | --- | --- | --- | --- | --- | --- | --- | --- | --- |
|  | AW | AWS | CB | CS | DD | DH | HB | LS | SF | SM |  |
| AWS | 0.069 |  |  |  |  |  |  |  |  |  |  |
| CB | 0.154 | 0.763 |  |  |  |  |  |  |  |  |  |
| CS | 0.396 | 0.044* | 0.154 |  |  |  |  |  |  |  |  |
| DD | 0.313 | 0.095 | 0.331 | 0.492 |  |  |  |  |  |  |  |
| DH | 0.092 | 0.812 | 0.889 | 0.044* | 0.127 |  |  |  |  |  |  |
| HB | 0.396 | 0.212 | 0.455 | 0.044* | 0.154 | 0.432 |  |  |  |  |  |
| LS | 0.044* | 0.044* | 0.154 | 0.044* | 0.127 | 0.044* | 0.044* |  |  |  |  |
| SF | 0.831 | 0.141 | 0.289 | 1.000 | 0.641 | 0.141 | 0.335 | 0.141 |  |  |  |
| SM | 0.529 | 0.082 | 0.180 | 0.044* | 0.212 | 0.154 | 1.000 | 0.044* | 0.450 |  |  |
| ZR | 0.044* | 0.113 | 0.044* | 0.044* | 0.044* | 0.141 | 0.069 | 0.044* | 0.095 | 0.044* |  |
| **Shannon p-values** | | | | | | | | | | | |
|  | AW | AWS | CB | CS | DD | DH | HB | LS | SF | SM |  |
| AWS | 0.022* |  |  |  |  |  |  |  |  |  |  |
| CB | 0.150 | 0.150 |  |  |  |  |  |  |  |  |  |
| CS | 0.503 | 0.022* | 0.889 |  |  |  |  |  |  |  |  |
| DD | 0.889 | 0.022* | 0.276 | 0.775 |  |  |  |  |  |  |  |
| DH | 0.079 | 0.022* | 0.889 | 0.503 | 0.118 |  |  |  |  |  |  |
| HB | 0.306 | 0.022* | 0.487 | 1.000 | 0.150 | 0.306 |  |  |  |  |  |
| LS | 0.022* | 0.022* | 0.150 | 0.022* | 0.118 | 0.022* | 0.022* |  |  |  |  |
| SF | 0.135 | 0.082 | 0.880 | 0.335 | 0.135 | 0.491 | 0.218 | 0.082 |  |  |  |
| SM | 0.022* | 0.022* | 0.276 | 1.000 | 0.150 | 0.405 | 0.641 | 0.022* | 0.655 |  |  |
| ZR | 0.022* | 0.022* | 0.150 | 0.022* | 0.022* | 0.022* | 0.022* | 0.022* | 0.135 | 0.022* |  |
| **Simpson p-values** | | | | | | | | | | | |
|  | AW | AWS | CB | CS | DD | DH | HB | LS | SF | SM |  |
| AWS | 0.029* |  |  |  |  |  |  |  |  |  |  |
| CB | 0.169 | 0.169 |  |  |  |  |  |  |  |  |  |
| CS | 0.426 | 0.029* | 0.873 |  |  |  |  |  |  |  |  |
| DD | 0.426 | 0.029* | 0.308 | 0.503 |  |  |  |  |  |  |  |
| DH | 0.089 | 0.029* | 0.655 | 0.873 | 0.169 |  |  |  |  |  |  |
| HB | 0.330 | 0.029* | 0.500 | 0.745 | 1.000 | 0.259 |  |  |  |  |  |
| LS | 0.745 | 0.029* | 0.169 | 0.641 | 0.330 | 0.133 | 0.169 |  |  |  |  |
| SF | 0.089 | 0.089 | 0.500 | 0.362 | 0.089 | 0.500 | 0.157 | 0.089 |  |  |  |
| SM | 0.055 | 0.029* | 0.308 | 0.745 | 0.873 | 0.330 | 0.503 | 0.029* | 0.157 |  |  |
| ZR | 0.029* | 0.029* | 0.169 | 0.029* | 0.029* | 0.029* | 0.029* | 0.029* | 0.500 | 0.029* |  |
