# Supplementary material for: Evaluating extraction methods to study canine urine microbiota
Source: PLoS One. 2021 Jul 9;16(7):e0253989. doi: 10.1371/journal.pone.0253989 (PMC8270191; doi:10.1371/journal.pone.0253989)
Supplement: S7 Table — P-values based on PERMANOVA pairwise comparisons using 1000 permutations False Discovery Rate corrections. *p < 0.05. (DOCX) [file pone.0253989.s012.docx]

**Table S7** – **Beta-diversity pairwise comparisons by dog**. P-values based on PERMANOVA pairwise comparisons using 1000 permutations False Discovery Rate corrections. * = p < 0.05

| **Bray Curtis** | | | | | | | | | | |
| --- | --- | --- | --- | --- | --- | --- | --- | --- | --- | --- |
|  | AW | AWS | CB | CS | DD | DH | HB | LS | SF | SM |
| AWS | 0.0227* |  |  |  |  |  |  |  |  |  |
| CB | 0.124 | 0.0582 |  |  |  |  |  |  |  |  |
| CS | 0.0227* | 0.0227* | 0.0561 |  |  |  |  |  |  |  |
| DD | 0.0227* | 0.0227* | 0.0561 | 0.0227* |  |  |  |  |  |  |
| DH | 0.0561 | 0.0227* | 0.0176 | 0.0227* | 0.0301* |  |  |  |  |  |
| HB | 0.284 | 0.0227* | 0.0561 | 0.0227* | 0.0227* | 0.0614 |  |  |  |  |
| LS | 0.0244* | 0.0227* | 0.0582 | 0.0227* | 0.0227* | 0.0227* | 0.0227* |  |  |  |
| SF | 0.0244* | 0.0244* | 0.106 | 0.0227* | 0.0227* | 0.0244* | 0.0301* | 0.0301* |  |  |
| SM | 0.0296* | 0.0227* | 0.0582 | 0.0227* | 0.0227* | 0.0227* | 0.0244* | 0.0227* | 0.0260* |  |
| ZR | 0.0227* | 0.0227* | 0.0582 | 0.0227* | 0.0227* | 0.0244* | 0.0227* | 0.0227* | 0.0244* | 0.0227* |
| **Unweighted UniFrac** | | | | | | | | | | |
|  | AW | AWS | CB | CS | DD | DH | HB | LS | SF | SM |
| AWS | 0.0335* |  |  |  |  |  |  |  |  |  |
| CB | 0.221 | 0.125 |  |  |  |  |  |  |  |  |
| CS | 0.0549 | 0.0336* | 0.0799 |  |  |  |  |  |  |  |
| DD | 0.148 | 0.0375* | 0.0779 | 0.0336* |  |  |  |  |  |  |
| DH | 0.200 | 0.0336* | 0.145 | 0.0387* | 0.0900 |  |  |  |  |  |
| HB | 0.515 | 0.0357* | 0.275 | 0.0336* | 0.125 | 0.761 |  |  |  |  |
| LS | 0.0335* | 0.0347* | 0.082 | 0.0336* | 0.0336 | 0.336 | 0.0336* |  |  |  |
| SF | 0.369 | 0.0549 | 0.515 | 0.0567 | 0.148 | 0.305 | 0.449 | 0.0387* |  |  |
| SM | 0.0335* | 0.0336* | 0.0848 | 0.0336* | 0.0336* | 0.0336* | 0.0530 | 0.0336* | 0.0387* |  |
| ZR | 0.114 | 0.0382* | 0.509 | 0.0336* | 0.0387* | 0.143 | 0.159 | 0.0336* | 0.197 | 0.0336* |
| **Weighted UniFrac** | | | | | | | | | | |
|  | AW | AWS | CB | CS | DD | DH | HB | LS | SF | SM |
| AWS | 0.0323* |  |  |  |  |  |  |  |  |  |
| CB | 0.150 | 0.0751 |  |  |  |  |  |  |  |  |
| CS | 0.0739 | 0.0376* | 0.0873 |  |  |  |  |  |  |  |
| DD | 0.425 | 0.0323* | 0.0833 | 0.0323* |  |  |  |  |  |  |
| DH | 0.859 | 0.0323* | 0.141 | 0.0419* | 0.299 |  |  |  |  |  |
| HB | 0.787 | 0.0323* | 0.141 | 0.0833 | 0.221 | 0.913 |  |  |  |  |
| LS | 0.849 | 0.0323* | 0.0789 | 0.0739 | 0.299 | 0.591 | 0.425 |  |  |  |
| SF | 0.787 | 0.0419* | 0.375 | 0.251 | 0.0484* | 0.598 | 0.787 | 0.0611 |  |  |
| SM | 0.0450* | 0.0323* | 0.100 | 0.0323* | 0.0323* | 0.0323* | 0.0323* | 0.0323* | 0.0484* |  |
| ZR | 0.426 | 0.0323* | 0.273 | 0.0366* | 0.0323* | 0.0323* | 0.0571 | 0.0323* | 0.0484* | 0.0323* |
