# Supplementary material for: Evaluating extraction methods to study canine urine microbiota
Source: PLoS One. 2021 Jul 9;16(7):e0253989. doi: 10.1371/journal.pone.0253989 (PMC8270191; doi:10.1371/journal.pone.0253989)
Supplement: S8 Table — Thirty-two taxa at the L7 level were differentially abundant by dog. (DOCX) [file pone.0253989.s013.docx]

**Table S8 – Differentially abundant taxa by dog**. Thirty-two taxa at the L7 level were differentially abundant by dog.

| **Taxa ID** | **W** |
| --- | --- |
| D_0__Bacteria;D_1__Firmicutes;D_2__Bacilli;D_3__Lactobacillales;D_4__Streptococcaceae;D_5__Streptococcus;D_6__ Streptococcus canis | 614 |
| D_0__Bacteria;D_1__Firmicutes;D_2__Bacilli;D_3__Lactobacillales;D_4__Streptococcaceae;D_5__Streptococcus;D_6__ Streptococcus halichoeri | 614 |
| D_0__Bacteria;D_1__Proteobacteria;D_2__Gammaproteobacteria;D_3__Pseudomonadales;D_4__Moraxellaceae;D_5__ Psychrobacter;D_6__Psychrobacter alimentarius | 614 |
| D_0__Bacteria;D_1__Proteobacteria;D_2__Gammaproteobacteria;D_3__Pasteurellales;D_4__Pasteurellaceae;D_5__Haemophilus;D_6__Pasteurellaceae bacterium canine oral taxon 272 | 614 |
| D_0__Bacteria;D_1__Proteobacteria;D_2__Gammaproteobacteria;D_3__Pseudomonadales;D_4__Pseudomonadaceae;D_5__ Pseudomonas;__ | 611 |
| D_0__Bacteria;D_1__Proteobacteria;D_2__Gammaproteobacteria;D_3__Pasteurellales;D_4__Pasteurellaceae;D_5__Haemophilus | 611 |
| D_0__Bacteria;D_1__Firmicutes;D_2__Bacilli;D_3__Bacillales;D_4__Family XII;D_5__Exiguobacterium;__ | 611 |
| D_0__Bacteria;D_1__Fusobacteria;D_2__Fusobacteriia;D_3__Fusobacteriales;D_4__Fusobacteriaceae;D_5__Fusobacterium;D_6__Fusobacterium sp. canine oral taxon 439 | 603 |
| D_0__Bacteria;D_1__Proteobacteria;D_2__Gammaproteobacteria;D_3__Betaproteobacteriales;D_4__Burkholderiaceae;D_5__ Massilia;__ | 603 |
| D_0__Bacteria;D_1__Tenericutes;D_2__Mollicutes;D_3__Mycoplasmatales;D_4__Mycoplasmataceae;D_5__Mycoplasma;D_6__Mycoplasma cynos C142 | 603 |
| D_0__Bacteria;D_1__Tenericutes;D_2__Mollicutes;D_3__Mycoplasmatales;D_4__Mycoplasmataceae;D_5__Mycoplasma;__ | 602 |
| D_0__Bacteria;D_1__Tenericutes;D_2__Mollicutes;D_3__Mycoplasmatales;D_4__Mycoplasmataceae;D_5__Ureaplasma;D_6__ Ureaplasma canigenitalium | 602 |
| D_0__Bacteria;D_1__Fusobacteria;D_2__Fusobacteriia;D_3__Fusobacteriales;D_4__Leptotrichiaceae;D_5__Oceanivirga;D_6__ uncultured bacterium | 600 |
| D_0__Bacteria;D_1__Firmicutes;D_2__Clostridia;D_3__Clostridiales;D_4__Peptococcaceae;D_5__Peptococcus;__ | 599 |
| D_0__Bacteria;D_1__Bacteroidetes;D_2__Bacteroidia;D_3__Bacteroidales;D_4__Prevotellaceae;D_5__Alloprevotella;D_6__ Prevotella sp. canine oral taxon 226 | 599 |
| D_0__Bacteria;D_1__Tenericutes;D_2__Mollicutes;D_3__Acholeplasmatales;D_4__Acholeplasmataceae;D_5__Acholeplasma;D_6__Mycoplasma feliminutum | 598 |
| D_0__Bacteria;D_1__Firmicutes;D_2__Erysipelotrichia;D_3__Erysipelotrichales;D_4__Erysipelotrichaceae;D_5__Solobacterium;D_6__uncultured Erysipelotrichaceae bacterium | 598 |
| D_0__Bacteria;D_1__Bacteroidetes;D_2__Bacteroidia;D_3__Bacteroidales;D_4__Tannerellaceae;D_5__Parabacteroides;__ | 597 |
| D_0__Bacteria;D_1__Firmicutes;D_2__Clostridia;D_3__Clostridiales;D_4__Lachnospiraceae;D_5__Acetitomaculum;D_6__ uncultured bacterium | 597 |
| D_0__Bacteria;D_1__Bacteroidetes;D_2__Bacteroidia;D_3__Sphingobacteriales;D_4__Sphingobacteriaceae;D_5__ Sphingobacterium;__ | 596 |
| D_0__Bacteria;D_1__Firmicutes;D_2__Bacilli;D_3__Bacillales;D_4__Family XI;D_5__Gemella;__ | 594 |
| D_0__Bacteria;D_1__Firmicutes;D_2__Bacilli;D_3__Lactobacillales;D_4__Enterococcaceae;D_5__Enterococcus;__ | 594 |
| D_0__Bacteria;D_1__Firmicutes;D_2__Clostridia;D_3__Clostridiales;D_4__Peptostreptococcaceae;D_5__Peptostreptococcus;__ | 593 |
| D_0__Bacteria;D_1__Proteobacteria;D_2__Gammaproteobacteria;D_3__Enterobacteriales;D_4__Enterobacteriaceae;D_5__ Escherichia-Shigella;__ | 584 |
| D_0__Bacteria;D_1__Proteobacteria;D_2__Alphaproteobacteria;D_3__Sphingomonadales;D_4__Sphingomonadaceae;D_5__ Novosphingobium;__ | 584 |
| D_0__Bacteria;D_1__Proteobacteria;D_2__Gammaproteobacteria;D_3__Enterobacteriales;D_4__Enterobacteriaceae;D_5__Pantoea | 581 |
| D_0__Bacteria;D_1__Bacteroidetes;D_2__Bacteroidia;D_3__Flavobacteriales;D_4__Flavobacteriaceae;D_5__Flavobacterium;D_6__Flavobacterium ummariense | 574 |
| D_0__Bacteria;D_1__Proteobacteria;D_2__Gammaproteobacteria;D_3__Enterobacteriales;D_4__Enterobacteriaceae;__;__ | 572 |
| D_0__Bacteria;D_1__Proteobacteria;D_2__Gammaproteobacteria;D_3__Betaproteobacteriales;D_4__Neisseriaceae;D_5__ Conchiformibius;D_6__Conchiformibius steedae | 570 |
| D_0__Bacteria;D_1__Bacteroidetes;D_2__Bacteroidia;D_3__Flavobacteriales;D_4__Weeksellaceae;D_5__Chryseobacterium;__ | 569 |
| D_0__Bacteria;D_1__Proteobacteria;D_2__Alphaproteobacteria;D_3__Rhizobiales;D_4__Devosiaceae;D_5__Devosia;__ | 561 |
| D_0__Bacteria;D_1__Proteobacteria;D_2__Gammaproteobacteria;D_3__Cardiobacteriales;D_4__Cardiobacteriaceae;D_5__ Suttonella;D_6__Rappaport israeli | 553 |
