# Supplementary material for: Evaluating extraction methods to study canine urine microbiota
Source: PLoS One. 2021 Jul 9;16(7):e0253989. doi: 10.1371/journal.pone.0253989 (PMC8270191; doi:10.1371/journal.pone.0253989)
Supplement: Attachment — Submitted filename: Revisions_Evaluating extraction methods to study canine urine microbiota.docx [file pone.0253989.s014.docx]

Evaluating Extraction Methods to Study Canine Urine Microbiota

Revisions

The authors have submitted a well written manuscript, describing an interesting study. Studies on the urinary microbiota are starting to get published, but so far, they have mostly been limited to humans. Even then, low DNA yields are common in these studies, and thus the protocols involved need to be improved further. I do, however, have a few comments on the manuscript.

**Abstract**

L. 19: insert may into the sentence – “ The urinary microbiota is the collection of microbes present in urine that MAY play a role in host health”, since this still needs to be proven.

**Introduction**

L. 51: change microbiome to microbiota

L. 50-52: delete the last part of the sentence “and distinct from contaminants”, since by the nature of your collection method, you will get a lot of contaminants and can therefore not rule this out.

L. 63: A recent paper on this topic has been published and should be referenced – Ammitzbøll et al., 2021. Pre- and postmenopausal women have different core urinary microbiota. Sci rep. 11(1):2212.

L. 78-79: “which are a valuable and translational model for human urinary tract diseases (54)” - This is a repeat of l. 69-70. Just delete it.

**Methods**

L. 96: “All urine samples were stored at -80 °C within 6 hours of urination”. How were they stored before this? 6 hours of room temperature storage can be critical for bacterial samples. This impact may be amplified due to the low biomass of bacteria in the urine samples.

Table 1. The authors describe that the QIAamp PowerFecal pro DNA kit only contains a thermal lysis step, but not mechanical lysis. However, manufacturers protocol for PowerFecal Pro DNA kit starts with adding the fecal sample to a lysis buffer (step 1), and vortexing fecal samples with beads for varying time based on the homogenizer used (step 2). As such, the kit combines chemical and mechanical lysis, but not thermal lysis. Based on the description of the protocol used in the current study, l. 163-172, the authors appear to replace the mechanical lysis included in the kit, with their own version of bead beating. This may have impacted the final DNA yield.

Table 1. continued: Similarly, the QIAamp PowerFecal DNA kit normally combines thermal lysis (step 4) with mechanical lysis similar to that of QIAamp PowerFecal Pro DNA Kit (step 5), but with another lysis buffer. The authors appear to have replaced the original mechanical lysis step with their own mechanical lysis and decided not to use the thermal lysis normally included in the protocol.

While optimization is critical, based on the modifications I am not sure whether it can be said that the authors have reliably tested the effects of these two DNA extraction kits for canine urinary microbiota DNA extraction. The authors have stated that they performed these modifications in the table text, but I will recommend mentioning it in the limitations section. Secondly, I believe the authors should correct the table text, to specify that the two kits do normally contain mechanical lysis. If the authors consider the bead beating implemented in the manufacturers protocol to be insufficient, they should include this as an argument for the modifications.

3 mL of urine is not a lot, especially considering the low DNA yield. While I understand that 30 mL may be a low volume from a small dog, 10 mL should be feasible.

Do you make replicate DNA extractions or is just one sample from each dog processed? Use of replicates would strengthen your study.

OTUs vs ASVs. For most of the methods and results sections, the authors refer to sequences using operational taxonomic units (OTUs), whereas from l. 352 they start to refer to the sequences as amplicon sequence variants (ASVs). Did the authors cluster the sample to OTUs, or did they use ASVs? I recommend being consistent with this.

L. 115-117: The naming of samples using the initials of the dogs is a bit confusing, especially given that you also use abbreviations for the five kits. I would recommend using numbers (e.g. 1-10 for the dogs).

**Results**

L. 253: Why do you have 12 samples here? Are there two positive controls? Later e.g. in line 284 you mention 11 samples (including a positive control). In general, it is difficult to follow your number of samples throughout the manuscript. Please be clearer on this.

L. 299: How did you determine what was considered contaminants? In lines 306-307 you state that two of your negative controls were contaminated by urine samples, but how can you determine this? Maybe reagent contaminants just tend to show up when the DNA concentrations are very low – which could be the case for both negative controls and urine samples. Should this influence your cut-off levels to be around 8200 instead of 300 reads? Please evaluate and comment on this.

L. 360-369 and again l. 463-479: How about genera? Often amplicon sequencing of the 16S rRNA gene focus on the taxonomic levels phylum, family, and genus (L6), and thus it would be interesting to hear if this was affected by extraction method.

**Discussion**

L. 419-420: Another example where the description is a bit confusing regarding the number of samples.

**Supplementary data**

S2 table. The font size is a bit small. The table can handle being a bit wider, which would free up space for an increased font size.
