# Supplementary material for: Evaluating extraction methods to study canine urine microbiota
Source: PLoS One. 2021 Jul 9;16(7):e0253989. doi: 10.1371/journal.pone.0253989 (PMC8270191; doi:10.1371/journal.pone.0253989)
Supplement: Attachment — Submitted filename: Response to reviewers.docx [file pone.0253989.s016.docx]

Dear Dr. Gyarmati and Reviewers,

We are pleased to share with you a second revision of our manuscript, *Evaluating Extraction Methods to Study Canine Urine Microbiota.* We thank the reviewers for their comments. We have addressed each of the suggestions point-by-point below in blue.

Please feel free to contact me if there is any further information you would like regarding this study.

Sincerely,

**Vanessa Hale MAT, DVM, PhD**

Co-Director, Infectious Disease Institute Microbial Communities Program

Associate Director, Center of Microbiome Science

Assistant Professor, Veterinary Preventive Medicine, College of Veterinary Medicine

Hale.502@osu.edu

6. Review Comments to the Author

Please use the space provided to explain your answers to the questions above. You may also include additional comments for the author, including concerns about dual publication, research ethics, or publication ethics. (Please upload your review as an attachment if it exceeds 20,000 characters)

Reviewer #1: Thank you for answering all questions and comments.

A few remarks:

- L. 248: "We extracted DNA from a total 10 dogs" - replace with "We extracted DNA from a total of 10 dogs".

Thank you for catching this error. The sentence has been edited.
- L. 500-502: Very long sentence and I think something has gone wrong in the sentence structure.

This sentence has been edited.
- L. 595-598: Use abbreviations for the kits.

We have now replaced kit names with abbreviations throughout the manuscript (except when we first introduce the kit names).
- Several places in the manuscript you switch between using the abbreviation and full name for the kits. Please be consistent.

We have now replaced kit names with abbreviations throughout the manuscript (except when we first introduce the kit names).

Reviewer #2: Manuscript Number: PONE-D-21-01761R1
Manuscript Title: Evaluating Extraction Methods to Study Canine Urine Microbiota
General comment: All the suggested comments have been addressed by the authors.
